# Increased Production of Ginsenoside Compound K by Optimizing the Feeding of American Ginseng Extract during Fermentation by *Aspergillus tubingensis*

**DOI:** 10.4014/jmb.2203.03059

**Published:** 2022-06-10

**Authors:** Woo-Seok Song, Min-Ju Kim, Kyung-Chul Shin, Deok-Kun Oh

**Affiliations:** 1 Department of Bioscience and Biotechnology, Konkuk University, Seoul 05029, Republic of Korea; 2 Department of Integrative Bioscience and Biotechnology, Konkuk University, Seoul 05029, Republic of Korea

**Keywords:** *Aspergillus tubingensis*, fermentation, compound K, protopanaxadiol-type ginsenosides, feeding optimization, American ginseng extract

## Abstract

The ginsenoside compound K (C-K) is widely used in traditional medicines, nutritional supplements, and cosmetics owing to its diverse pharmacological activities. Although many studies on C-K production have been conducted, fermentation is reported to produce C-K with low concentration and productivity. In the present study, addition of an inducer and optimization of the carbon and nitrogen sources in the medium were performed using response surface methodology to increase the C-K production via fermentation by *Aspergillus tubingensis*, a generally recognized as safe fungus. The optimized inducer and carbon and nitrogen sources were 2 g/l rice straw, 10 g/l sucrose, and 10 g/l soy protein concentrate, respectively, and they resulted in a 3.1-fold increase in the concentration and productivity of C-K (0.22 g/l and 1.52 mg/l/h, respectively) compared to those used before optimization without inducer (0.071 g/l and 0.49 mg/l/h, respectively). The feeding methods of American ginseng extract (AGE), including feeding timing, feeding concentration, and feeding frequency, were also optimized. Under the optimized conditions, *A. tubingensis* produced 3.96 mM (2.47 g/l) C-K at 144 h by feeding two times with 8 g/l AGE at 48 and 60 h, with a productivity of 17.1 mg/l/h. The concentration and productivity of C-K after optimization of feeding methods were 11-fold higher than those before the optimization (0.22 g/l and 1.52 mg/l/h, respectively). Thus, the optimization for the feeding methods of ginseng extract is an efficient strategy to increase C-K production. To our knowledge, this is the highest reported C-K concentration and productivity via fermentation reported so far.

## Introduction

Ginseng (*Panax ginseng* C. A. Meyer) has been used as a traditional herbal medicine in East Asia for more than a thousand years to strengthen immunity and reduce fatigue. Ginsenosides, the main biologically active compounds of ginseng, are widely used in food, cosmetic, and pharmaceutical industries due to their pharmacological effects, such as anti-allergy [1], anti-cancer [2], anti-fatigue [3], anti-inflammatory [4], anti-oxidative [5], and anti-stress properties [6]. Ginsenosides are divided into protopanaxadiol (PPD)-type and protopanaxatriol (PPT)-type ginsenosides according to the number and position of hydroxyl groups attached to the triterpenoid. They contain glycosides, such as D-glucopyranoside, D-xylopyranoside, L-arabinopyranoside, L-arabinofuranoside, and L-rhamnopyranoside, which are attached to C-3, C-6, and C-20 in PPD and PPT aglycons via glycosidic bonds.

The major ginsenosides are Rb1, Rb2, Rc, Rd, Re, and Rf, which make up more than 80% of the ginsenosides in natural ginseng. Minor ginsenosides, namely F1, Rg3, Rh1, Rh2, and compound K (C-K), which are deglycosylated from major ginsenosides, are not present in natural ginseng or present in very low concentrations [7]. In the gastrointestinal tract, the major ginsenosides with relatively larger molecular sizes are poorly absorbed, whereas the minor ginsenosides with smaller molecular sizes and higher cell membrane permeability are easily absorbed into the intestinal cell membrane and exhibit higher bioavailability than major ginsenosides [8].

To increase the absorption rate of major ginsenosides, the sugar moiety must be hydrolyzed and converted to minor ginsenosides. Therefore, many studies using physical, chemical, and biological methods to produce minor ginsenosides from major ginsenosides have been conducted [9]. Among these methods, physical and chemical methods have several limitations, such as low selectivity, generation of by-products, non-environment friendly, and high energy consumption. To overcome these disadvantages, biological methods, including enzyme conversion, cell conversion, and fermentation, have been utilized [10]. Although enzyme transformation shows higher conversion and productivity, it is less economical than fermentation because it requires enzyme purification, which results in the loss of enzymes during the purification from cells [11]. On the other hand, fermentation is relatively cost-effective because it does not require enzyme purification steps and can use both intracellular and extracellular enzymes.

The minor PPD-type ginsenoside C-K is one of the most biologically active ginsenosides [12, 13]. C-K is produced via enzyme conversion, cell conversion, and fermentation [10]. C-K production via fermentation shows significantly lower productivity than enzyme conversion [14-21]. To overcome this limitation of fermentation, a new strategy, such as feeding optimization of ginseng extract is required.

In this study, we optimized the inducer and carbon and nitrogen sources in the fermentation medium to increase C-K production by *Aspergillus tubingensis*, a generally recognized as safe (GRAS) fungus. The concentration and productivity of C-K were also increased by optimizing the feeding with American ginseng extract (AGE) during fermentation.

## Materials and Methods

### Materials

The ginsenoside standards (≥ 98% purity), including Rb1, Rb2, Rc, Rd, F2, compound Mc1 (C-Mc1), compound Mc (C-Mc), compound O (C-O), compound Y (C-Y), and C-K, were purchased from Ambo Institute (Republic of Korea). AGE was provided by Ace EMzyme (Republic of Korea). AGE contained 38.5% (w/w) PPD-type ginsenosides, which consist of Rb1 (204 mg/g), Rb2 (12 mg/g), Rc (74 mg/g), and Rd (95 mg/g). Korean ginseng extract contained 46.4% (w/w) PPD-type ginsenosides, which consisted of Rb1 (165 mg/g), Rb2 (117 mg/g), Rc (91 mg/g), and Rd (91 mg/g) [20]. The extracellular enzyme from *A. tubingensis* KCTC 14166 showed low hydrolytic activity for Rb2. We used AGE as a substrate for C-K production by *A. tubingensis* KCTC 14166 because of its lower content of Rb2. A sucrose assay kit was purchased from BioAssay Systems (USA).

### Fungal Strain, Media, and Fermentation Conditions

The fungus *A. tubingensis* KCTC 14166 (Korean Collection for Type Cultures, Republic of Korea) was used for C-K production [20]. *A. tubingensis* was incubated on potato dextrose agar for 14 days. Thereafter, distilled water mixed with 0.1% (v/v) Triton-X_1_00 was added to the plate. Spores were collected by raking agar surface using a spreader and the obtained spore solution was filtered using two layers of gauze and diluted to 1.0 × 10^6^ spores/ml. The number of spores was counted using a hemocytometer (INCYTO, Republic of Korea). One ml of the spore suspension was inoculated into 5 ml of potato dextrose broth (PDB) in glass tube and incubated for 24 h at 28°C with shaking at 150 rpm. Subsequently, the grown mycelia were inoculated into a 500 ml-baffled Erlenmeyer flask containing 100 ml of the fermentation medium and incubated for 144 h at 28°C with shaking at 150 rpm. The fermentation medium before the optimization of inducer and carbon and nitrogen sources contained 20 g/l citrus pectin, 10 g/l corn steep solid, 5 g/l KH_2_PO_4_, 5 g/l Na_2_HPO_4_, 0.3 g/l MgSO_4_·7H_2_O, 0.3 g/l CaCl_2_, 5 mg/l FeSO_4_·7H_2_O, and 1.3 mg/l MnSO_4_·H_2_O. The pH of fermentation media was adjusted to 5.0. Unless otherwise stated, fermentation was conducted in a shaking incubator at 28°C and 150 rpm for 144 h, and 1 g/l AGE containing 0.36 mM PPD-type ginsenosides as the substrate for C-K production was added at 72 h.

### Optimization of the Inducer and Carbon and Nitrogen Sources

The effect of inducer on C-K production during fermentation of *A. tubingensis* was investigated using 2 g/l of arabinogalactan, arabic gum, grapefruit pectin, apple pectin, rice straw, sugar beet sludge, and sugar beet fiber. The effect of carbon source on C-K production was tested using 20 g/l of citrus pectin, cellulose, fructose, sucrose, lactose, and wheat bran, supplemented with 2 g/l rice straw. The effect of nitrogen source on C-K production was evaluated using 10 g/l of corn steep solid, soybean powder, soy protein concentrate, yeast extract, and urea supplemented with 2 g/l rice straw.

The optimized fermentation medium factors for C-K production were proposed through Box-Behnken design (BBD) of response surface methodology (RSM) and analyzed by Design Expert 10.0.2 (Stat-Ease, USA). The three independent variables for C-K production were labeled and coded as follow: inducer concentration, X_1_ (-1, 0, and 1 as 1, 2, and 3 g/l); sucrose concentration, X_2_ (-1, 0, and 1 as 5, 10, and 20 g/l); and soy protein concentrate concentration, X_3_ (-1, 0, and 1 as 5, 10, and 20 g/l). The reactions for optimizing the concentrations were performed at pH 5.0 and 28 °C.

### Optimization of Feeding Methods for AGE

To determine the optimal feeding timing of AGE as a substrate for C-K production, 1 g/l AGE was fed one time in the optimized fermentation medium at 24–84 h during fermentation. The optimal feeding concentration of AGE at 60 h was determined using varying the AGE concentrations from 1 to 16 g/l). To reduce the toxicity towards the fungus and the inhibition of C-K production caused by the high concentration of AGE, feeding was performed two or three times at 48, 60, or/and 72 h during fermentation.

### Production of C-K via Fermentation under Optimized Conditions

C-K production via fermentation by *A. tubingensis* was performed under the optimized feeding conditions of feeding two times with 8 g/l AGE at 48 and 60 h, with a total of 16 g/l AGE containing 5.76 mM or 6.16 g/l PPD-type ginsenosides to the optimized fermentation medium containing 2 g/l rice straw, 10 g/l sucrose, 10 g/l soy protein concentrate, 5 g/l KH_2_PO_4_, 5 g/l Na_2_HPO_4_, 0.3 g/l MgSO_4_·7H_2_O, 0.3 g/l CaCl_2_, 5 mg/l FeSO_4_·7H_2_O, and 1.3 mg/l MnSO_4_·H_2_O with shaking at 150 rpm for 156 h at 28°C.

### Analytical Methods

After fermentation, the fermentation broth was extracted by adding an equal volume of *n*-butanol mixed with 1.0 mg/ml digoxin as an internal standard. The *n*-butanol layer was separated and collected via centrifugation at 13,000 ×*g* for 10 min. The collected layer was evaporated until it was completely dried, and an equal volume of methanol was added to the dried residue. Ginsenosides dissolved in methanol were quantitatively analyzed using the Agilent 1100 Infinity HPLC system with a UV detector at 203 nm and an octadecylsilica column (4.6 × 150 mm, 5 μm particle size; YMC, Japan). The column was eluted at 40°C with a gradient of acetonitrile/water (v/v) from 30:70 to 60:40 for 20 min, 60:40 to 90:10 for 10 min, 90:10 to 30:70 for 5 min, and 30:70 for 10 min, at a flow rate of 1 ml/min. All ginsenosides, including reagent ginsenoside standards, ginsenosides in AGE, and biotransformed ginsenosides in AGE, were quantified by the calibration curves using the ginsenoside standards. The fermentation broth was filtered with Whatman No. 1 filter paper and washed with water, and the collected cells were dried in a dry oven at 105°C for 12 h. After drying, the dry cell weight was measured.

### Statistical Analysis

The means and standard errors for all experiments, including evaluation of fermentation conditions, and HPLC analysis, were calculated from duplicate. One-way analysis of variance (ANOVA) was carried out using Tukey’s method with a significance level of *p* < 0.05 using SigmaPlot 10.0 (Systat Software, USA).

## Results and Discussion

### Effect of Inducer on C-K Production via Fermentation by *A. tubingensis*

**Fig. 1:**
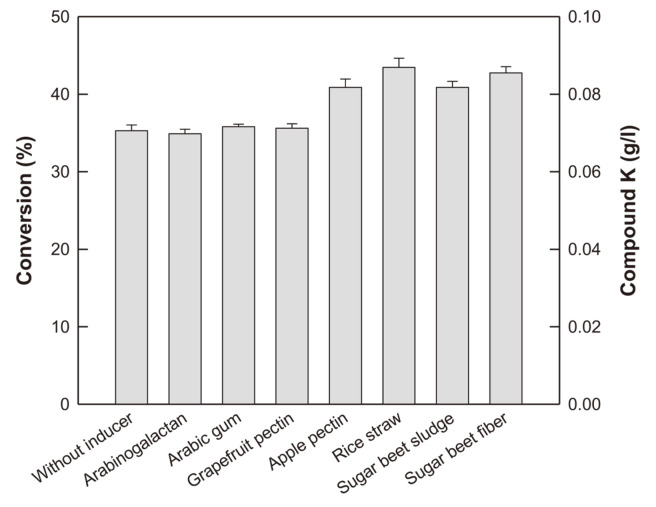
Effect of inducer on C-K production by fermentation by *A. tubingensis*. Different polysaccharides at 2 g/l were added as inducers. Data were examined in duplicate, and the error bar represents the standard deviation.

The extracellular enzyme from *A. tubingensis* KCTC 14166 with D-glucosidase, L-arabinofuranosidase, and L-arabinopyranosidase activities showed the highest concentration and productivity of C-K among the enzymatic transformation reported so far [20]. Polysaccharides used in fungal cultivation are known to induce the synthesis of hydrolytic enzymes [22] and increase their activity [23]. Therefore, in the present study, C-K production via fermentation by *A. tubingensis* KCTC 14166 was evaluated by adding 1 g/l AGE at 72 h with 2 g/l polysaccharide as an inducer in the medium containing 20 g/l citrus pectin and 10 g/l corn steep solid, as carbon and nitrogen sources, respectively. Among the polysaccharides, rice straw produced C-K with the highest concentration (0.087 g/l) and molar conversion of PPD-type ginsenosides in AGE to C-K (36.2%), which were 123% higher than those (0.071 g/l and 29.4%, respectively) without polysaccharides (Fig. 1). Therefore, 2 g/l rice straw was supplemented in the fermentation medium for effective C-K production.

### Effects of Carbon and Nitrogen Sources on C-K Production via Fermentation by *A. tubingensis*

**Fig. 2:**
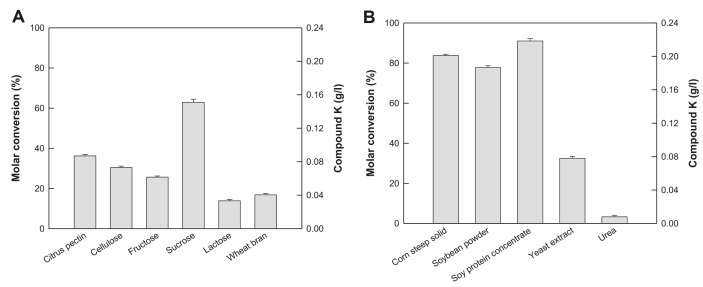
Effects of carbon and nitrogen source on C-K production by fermentation by *A. tubingensis*. (**A**) Effect of carbon source. (**B**) Effect of nitrogen source. The substrate AGE at 1 g/l was added at 72 h. Data were examined in duplicate, and the error bar represents the standard deviation.

Carbon sources have been used to increase the synthesis of hydrolytic enzymes during fungal cultivation [24]. To select the optimal carbon source, 20 g/l of various carbon sources were added into the medium containing 10 g/l corn steep solid and 2 g/l rice straw (Fig. 2A). Sucrose was identified as the optimal carbon source because it led to the highest production of C-K.

The activity of hydrolytic enzymes increases by optimizing the nitrogen source during fermentation [25]. Therefore, C-K production was investigated using 10 g/l of various nitrogen sources in the medium containing 20 g/l sucrose and 2 g/l rice straw. C-K production was the highest using soy protein concentrate (Fig. 2B).

The optimization of the concentrations of inducer, sucrose, and soy protein concentrate for C-K production during fermentation by *A. tubingensis* was performed using RSM. BBD of RSM for the three independent variables, rice straw concentration (X_1_), sucrose concentration (X_2_), and soy protein concentrate concentration (X_3_) for C-K production was made with 17 experimental runs (Table 1). The final polynomial equations derived from regression equations using analysis of variance (ANOVA) in terms of coded and actual factors, which were represented by the following equations : Y_1 coded_ = +0.2217 + 0.0001X_1_ – 0.0071X_2_ – 0.0063X_3_ + 0.0021X_1_X_2_ –0.0031X_1_X_3_ -0.0123X_2_X_3_ – 0.0312X_1_^2^ – 0.0303X_2_^2^ – 0.0248X_3_^2^ (1) and Y_1 actual_ = +0.221709 + 0.000117X_1_ –0.007107X_2_ – 0.006307X_3_ + 0.002145X_1_X_2_ – 0.003059X_1_X_3_ – 0.012285X_2_X_3_ – 0.031226X_1_^2^ – 0.030303X_2_^2^ –0.024810X_3_^2^ (2), respectively. The ANOVA for the model and factor is indicated in Table 2. The probability value (P value) indicated the significance of the interaction between each independent variable. The regression model was statistically significant (*p* < 0.05), meaning that it could accurately predict the results of ANOVA for each variable (rice straw, sucrose, and soy protein concentrate concentration). According to the ANOVA, the regression coefficient (R^2^) of the model was calculated to be 99.6%, indicating that the model had adequately represented the real relationships between the parameters chosen.

**Fig. 3:**
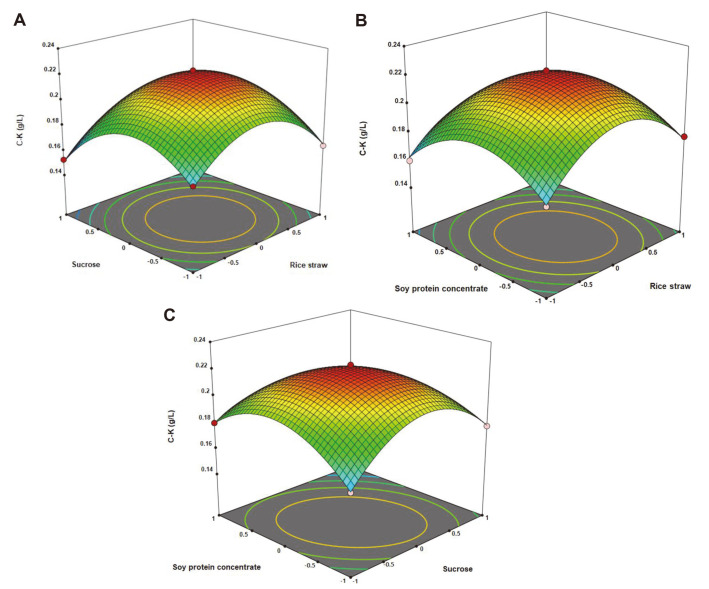
Response surface plots of rice straw, sucrose, and soy protein concentrate concentration for C-K production. (**A**) Plot of sucrose and rice straw concentration. (**B**) Plot of soy protein concentrate and rice straw concentration. (**C**) Plot of soy protein concentrate and sucrose concentration. The substrate AGE 1 g/l was added at 72 h.

The interactive relationships between the concentrations of rice straw, sucrose, and soy protein concentrate for C-K production are represented using three-dimensional contour plots in Fig. 3. The optimal values of the three variables in coded units were X_1_ = 0, X_2_ = 0, and X_3_ = 0. Therefore the optimal concentrations of rice straw, sucrose, and soy protein concentrate for C-K production during fermentation by *A. tubingensis* were 2 g/l rice straw, 10 g/l sucrose, and 10 g/l soy protein concentrate, respectively.

C-K production (0.22 g/l) and molar conversion (91.0%) using 2 g/l rice straw, 10 g/l sucrose and 10 g/l soy protein concentrate as the optimal inducer and carbon and nitrogen sources, respectively, were increased by 2.5- and 2.1-fold compared to the C-K production (0.087 g/l) and molar conversion (43.5%) using 20 g/l citrus pectin and 10 g/l corn steep solid before the optimization. These results demonstrate that the inducer and the types and concentrations of the carbon and nitrogen sources are important for increasing C-K production by *A. tubingensis*. Moreover, corn steep solid is limited as a food source, whereas soy protein concentrate is not limited.

### Effects of Feeding Timing and Concentration of AGE on C-K Production via Fermentation by *A. tubingensis*

**Fig. 4:**
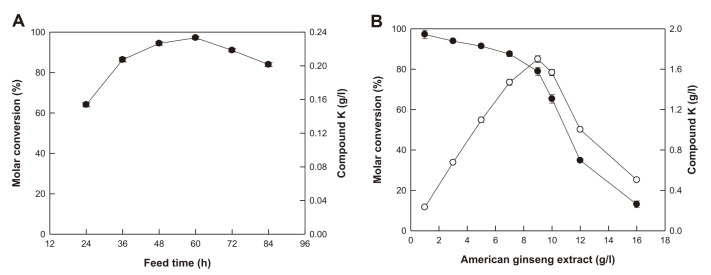
Effects of feeding timing and AGE concentration on C-K production by fermentation by *A. tubingensis*. (**A**) Effect of feeding timing. AGE as a substrate for C-K production at 1 g/l was added at each feeding timing from 24 to 84 h. (**B**) Effect of AGE concentration. ●, conversion; ○, C-K concentration. Data were examined in duplicate, and the error bar represents the standard deviation.

To optimize the feeding timing of AGE as a substrate for C-K production, 1 g/l AGE was added to fermentation broth at different timepoints from 24 to 84 h (Fig. 4A). C-K production was the highest by feeding at 60 h, with a molar conversion of 97.2%. Subsequently, the effect of AGE concentration on C-K production at 60 h of feeding timing was investigated by varying the concentration from 1 to 16 g/l (Fig. 4B). An increase in AGE concentration resulted in a decrease in conversion, whereas an increase in AGE concentration within 9 g/l led to a proportional increase in C-K production. However, above 9 g/l AGE, the C-K production decreased with increasing AGE concentration. These results indicate that the optimal AGE concentration at the optimal feeding timing is 9 g/l.

### Effect of Feeding Frequency of AGE on C-K Production via Fermentation by *A. tubingensis*

When a high concentration of substrate has a negative effect, it can be overcome via intermittent feeding [26]. Intermittent feeding was performed by feeding AGE two to three times to increase C-K concentration and productivity. Table 3 shows the C-K production during intermittent feeding and AGE concentration during fermentation. The optimal feeding timings were selected to be 48, 60, and 72 h because the molar conversion was the highest during feeding at 60 h and was > 90% during feeding at 48 and 72 h (Fig. 4A). The molar conversion, concentration, and productivity of C-K obtained from feeding two times with 4.5 g/l AGE at 48 and 60 h, with a total of 9 g/l AGE (Run 8 of Table 3), were 110% higher than those fed one time with 9 g/l AGE at 60 h (Fig. 4B). When 8 g/l AGE was fed two times at 48 and 60 h, with a total concentration of 16 g/l (Run 14), C-K concentration (2.47 g/l) and productivity (17.1 mg/ l /h) were the highest among those obtained by feeding of AGE. This feeding type resulted in a 4.9-fold increase in the concentration and productivity compared to feeding one time with 16 g/l AGE. The concentration and productivity of C-K after optimizing the feeding increased 11-fold compared to those before optimization (0.22 g/l and 1.52 mg/ l / h, respectively).

C-K production by feeding three times with AGE at 48, 60, and 72 h was also examined. When a total of more than 16 g/l AGE was fed three times, C-K production was reduced compared to feeding two times. For instance, when a total of 16 g/l AGE was added three times (6 g/l AGE at 48 h, 6 g/l AGE at 60 h, and 4 g/l AGE at 72 h), the concentration and productivity of C-K were reduced to 94.7% compared to those obtained by feeding two times with 8 g/l AGE at 48 and 60 h. Meanwhile, the concentration and productivity of C-K fed with a total of 24 g/l AGE three times (8 g/l AGE at 48, 60, and 72 h) were reduced to 42% compared to those obtained by feeding two times with 12.5 g/l AGE at 48 and 60 h. These results indicate that feeding two times is more effective for C-K production than feeding one time and three times. Moreover, feeding with 8 g/l AGE at 48 and 60 h is optimal for C-K production.

### C-K Production from PPD-Type Ginsenosides in AGE via Fermentation by *A. tubingensis* under Optimized Conditions

**Fig. 5:**
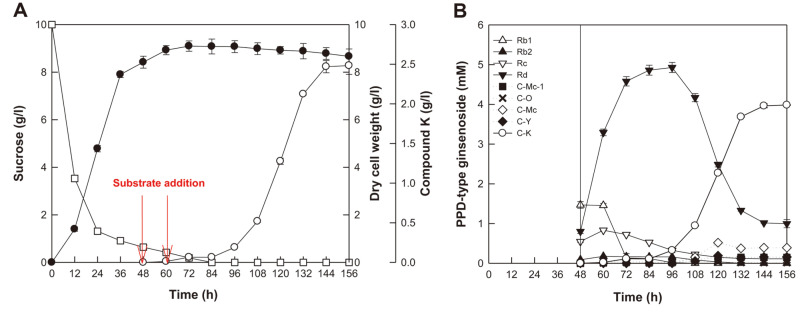
Production of C-K from PPD-type ginsenosides in AGE by fermentation by *A. tubingensis*. (**A**) Mycelial growth and C-K production by fermentation by *A. tubingensis*. Red arrows represent the feeding of 8 g/l AGE. ●, dry cell weight; □, sucrose concentration; ○, C-K concentration. (**B**) Biotransformation of PPD-type ginsenosides in AGE into C-K via the intermediates C-Mc1, C-O, C-Mc, and C-Y by fermentation by *A. tubingensis*. The vertical line at 48 h represents beforeand after-feeding periods of AGE. Data were examined in duplicate, and the error bar represents the standard deviation.

AGE contained 38.5% (w/w) PPD-type ginsenosides, which consisted of Rb1 (204 mg/g), Rb2 (12 mg/g), Rc (74 mg/g), and Rd (95 mg/g). Fermentation for the biotransformation of PPD-type ginsenosides in AGE to C-K was performed for 156 h in the optimized fermentation medium with shaking at 150 rpm at 28°C, with feeding two times with 8 g/l AGE at 48 h and 60 h. After 144 h of fermentation, C-K production reached a plateau (Fig. 5A). *A. tubingensis* produced 2.47 g/l C-K at 144 h, with a molar conversion of 64.3%, productivity of 17.1 mg/l/h, and dry cell weight of 8.78 g/l. The extracellular enzymes of *A. tubingensis* showed glycoside-hydrolyzing pathways as follows: Rb1 → Rd → F2 → C-K, Rb2 → C-O or Rd → C-Y or F2 → C-K, and Rc → Rd or C-Mc1 → F2 or C-Mc → C-K [20]. Rb1, Rb2, and Rc in AGE supplied at 48 and 60 h were converted into Rd, which accumulated to approximately 5 mM at 96 h (Fig. 5B) and the accumulated Rd was converted into C-K via F2. Rb2 and Rc in AGE were converted into C-K via C-O and C-Y; and C-Mc1 and C-Ms using alternative pathways, respectively. During the fermentation by *A. tubingensis*, further hydrolysis of the formed C-K was not found. After 144 h, the residual concentrations of byproduct ginsenosides followed the order Rd (1.0 mM) > C-Mc (0.39 mM) > C-Y (0.15 mM) > C-Mc1 (0.14 mM) > C-O (0.02 mM). These results indicate that the hydrolysis of Rc to C-K via C-Mc1 and C-Mc and that of Rb2 to C-K via C-O and C-Y via fermentation by *A. tubingensis* are incomplete.

The production of C-K from PPD-type ginsenosides in the ginseng extracts via fermentation is shown in Table 4. *Paecilomyces bainier* sp. 229 produced 1.25 g/l C-K via fermentation for 144 h, with a productivity of 8.6 mg/l/h and a molar conversion of 82.6%, all of which were the previously highest recorded values for C-K [15]. The concentration (2.47 g/l) and productivity (17.1 mg/l/h) of C-K obtained via fermentation by *A. tubingensis* under the optimized conditions were approximately 2-fold higher than those of *P. bainier* sp. 229. *A. tubingensis* also produced 1.87 g/l C-K at 144 h, with a productivity of 12.9 mg/l/h and a molar conversion of 86.4%. These results showed a higher concentration, productivity, and conversion than those of *P. bainier* sp. 229. Among the GRAS fungi, *Aspergillus niger* FMBS 494 produced the highest concentration (0.04 g/l) and productivity (0.625 mg/l/h) of C-K [19], which were 62-, and 27-fold lower than those obtained in the present study. These results indicate that *A. tubingensis* is a potential C-K-producer, and optimization of feeding with ginseng extract is an effective tool for increasing C-K production.

In conclusion, we optimized the inducer and carbon and nitrogen sources in the fermentation medium; and the AGE feeding timing, concentration, and frequency to increase C-K production via fermentation by *A. tubingensis*. Under the optimized conditions, *A. tubingensis* effectively produced food-available C-K from PPD-type ginsenosides in AGE. To the best of our knowledge, this is the highest concentration, productivity, and conversion of C-K among those produced via fermentation to date. Moreover, since *A. tubingensis* was a GRAS fungus, this study may be helpful in the industrial production of food-available C-K.

## Figures and Tables

**Table 1:** Experimental design for three independent variables by RSM and the production of C-K during fermentation by *A. tubingensis*.

Run	Factors^a^			C-K (g/l)

	X_1_	X_2_	X_3_	
1	−1	−1	0	0.171
2	1	-1	0	0.164
3	−1	1	0	0.153
4	1	1	0	0.154
5	−1	0	−1	0.168
6	1	0	−1	0.177
7	−1	0	1	0.160
8	1	0	1	0.157
9	0	−1	−1	0.167
10	0	1	−1	0.177
11	0	−1	1	0.180
12	0	0	1	0.193
13	0	0	0	0.218
14	0	0	0	0.223
15	0	0	0	0.222
16	0	0	0	0.221
17	0	0	0	0.222

^a^X_1_, rice straw concentration; X_2_, sucrose concentration; X_3_, soy protein concentrate concentration

**Table 2:** ANOVA results for the response surface quadratic mode.

Term	Sum of squares	df	Mean square	*F* value	*P* value
Model	0.0115	9	0.0013	222.64	<0.0001
X_1_	1.098E-07	1	1.098E-07	0.0191	0.8939
X_2_	0.0003	1	0.0003	49.49	0.0002
X_3_	0.0003	1	0.0003	46.59	0.0002
X_1_ × X_2_	0.0000	1	0.0000	3.21	0.0002
X_1_ × X_3_	0.0000	1	0.0000	6.52	0.0379
X_2_ × X_3_	0.0003	1	0.0003	57.01	0.0001
X_1_^2^	0.0037	1	0.0037	640.95	<0.0001
X_2_^2^	0.0031	1	0.0031	533.43	<0.0001
X_3_^2^	0.0024	1	0.0024	416.81	<0.0001
Residual	0.0000	7	5.740E-06	−	−
Lack of fit	0.0000	3	8.489E-06	2.31	0.2182
Pure error	0.0000	4	3.678E-06	−	−
Cor total	0.0115	16	−	−	−

X_1_, rice straw concentration; X_2_, sucrose concentration; X_3_, soy protein concentrate concentration; df, degrees of freedom; *R*^2^ = 0.9965; *R*^2^ (adj) = 0.9920; *R*^2^ (pred) = 0.9444.

**Table 3:** Effect of feeding two times with AGE on C-K production during fermentation by *A. tubingensis*.

Run	Feeding timing			Total AGE (g/l)	Molar conversion (%)	C-K (g/l)	Productivity (mg/l/h)

	48 h	60 h	72 h				

	AGE (g/l)						
1	1	1	0	2	98.2 ± 1.34	0.47 ± 0.01	3.26 ± 0.04
2	2	2	0	4	95.7 ± 1.91	0.92 ± 0.02	6.38 ± 0.12
3	0	2	2	4	94.1 ± 2.33	0.90 ± 0.02	6.27 ± 0.15
4	3	3	0	6	90.6 ± 1.57	1.30 ± 0.02	9.06 ± 0.14
5	0	3	3	6	87.3 ± 0.94	1.26 ± 0.01	8.73 ± 0.08
6	4	4	0	8	86.3 ± 1.19	1.66 ± 0.02	11.5 ± 0.14
7	0	4	4	8	85.6 ± 1.28	1.64 ± 0.02	11.4 ± 0.15
8	4.5	4.5	0	9	86.4 ± 2.10	1.87 ± 0.05	12.9 ± 0.16
9	0	4.5	4.5	9	85.3 ± 2.30	1.84 ± 0.05	12.7 ± 0.17
10	5	5	0	10	83.2 ± 1.40	1.99 ± 0.03	13.8 ± 0.12
11	0	5	5	10	81.6 ± 1.50	1.95 ± 0.04	13.6 ± 0.12
12	6	6	0	12	81.3 ± 0.90	2.34 ± 0.03	16.2 ± 0.09
13	7	7	0	14	71.4 ± 1.10	2.39 ± 0.04	16.6 ± 0.13
14	8	8	0	16	64.3 ± 1.40	2.47 ± 0.05	17.1 ± 0.19
15	9	9	0	18	55.5 ± 2.00	2.39 ± 0.09	16.6 ± 0.30
16	10	10	0	20	44.2 ± 1.70	2.12 ± 0.08	14.7 ± 0.28
17	12.5	12.5	0	25	37.5 ± 3.11	2.25 ± 0.07	15.6 ± 0.49
18	15	15	0	30	30.2 ± 2.96	2.17 ± 0.06	15.1 ± 0.45
19	6	6	4	16	60.8 ± 2.31	2.33 ± 0.09	16.2 ± 0.42
20	6	6	6	18	51.8 ± 1.69	2.24 ± 0.07	15.5 ± 0.31
21	7	7	6	20	25.8 ± 2.41	1.24 ± 0.12	8.61 ± 0.40
22	8	7	7	22	25.3 ± 1.98	1.34 ± 0.10	9.27 ± 0.42
23	8	8	8	24	16.4 ± 3.11	0.94 ± 0.18	6.56 ± 0.02

**Table 4:** Production of C-K from ginseng extract by fermentation using fungi.

Fungus	Ginseng extract	C-K (g/l)	Molar conversion (%)	Productivity (mg/l/h)	Reference
*Fusarium sacchari*	Saponins of *Panax notoginseng* extract	0.25	24	1.74	[14]
*Paecilomyces bainier* sp. 229	Saponins of *Panax notoginseng* leaves	1.25	82.6	8.6	[15]
*Ganoderma lucidum* CRC 37066	American ginseng root extraction residue	0.005	3.0	0.01	[17]
*Aspergillus niger* KACC 46494	Korean ginseng berry extract	NC	10.3	NC	[18]
*Aspergillus oryzae* KACC 40247		NC	3.4	NC	
*Aspergillus niger* FMBS 494	Korean ginseng	0.04	66.8	0.625	[19]
*Cordyceps sinensis*	Red ginseng extract	0.11	NC	NC	[16]
*Aspergillus tubingensis* KCTC 14166	American ginseng extract	1.87	86.4	12.9	This study
		2.47	64.3	17.1	

NC: Not calculated.

## References

[1] Han MJ, Kim DH (2020). Effects of red and fermented ginseng and ginsenosides on allergic disorders. Biomolecules.

[2] Yin Q, Chen H, Ma RH, Zhang YY, Liu MM, Thakur K (2021). Ginsenoside CK induces apoptosis of human cervical cancer HeLa cells by regulating autophagy and endoplasmic reticulum stress. Food Funct..

[3] Oh HA, Kim DE, Choi HJ, Kim NJ, Kim DH (2015). Anti-fatigue effects of 20(S)-protopanaxadiol and 20(S)-protopanaxatriol in mice. Biol. Pharm. Bull..

[4] Lee SY, Jeong JJ, Eun SH, Kim DH (2015). Anti-inflammatory effects of ginsenoside Rg1 and its metabolites ginsenoside Rh1 and 20(S)-protopanaxatriol in mice with TNBS-induced colitis. Eur. J. Pharmacol..

[5] Han Y, Wang T, Li C, Wang Z, Zhao Y, He J (2021). Ginsenoside Rg3 exerts a neuroprotective effect in rotenone-induced Parkinson's disease mice via its anti-oxidative properties. Eur. J. Pharmacol..

[6] Oh HA, Kim DE, Choi HJ, Kim NJ, Kim DH (2015). Anti-stress effects of 20(S)-protopanaxadiol and 20(S)-protopanaxatriol in immobilized mice. Biol. Pharm. Bull..

[7] Park CS, Yoo MH, Noh KH, Oh DK (2010). Biotransformation of ginsenosides by hydrolyzing the sugar moieties of ginsenosides using microbial glycosidases. Appl. Microbiol. Biotechnol..

[8] Xu QF, Fang XL, Chen DF (2003). Pharmacokinetics and bioavailability of ginsenoside Rb1 and Rg1 from *Panax notoginseng* in rats. J. Ethnopharmacol..

[9] Zheng MM, Xu FX, Li YJ, Xi XZ, Cui XW, Han CC (2017). Study on transformation of ginsenosides in different methods. Biomed Res. Int..

[10] Shin KC, Oh DK (2016). Classification of glycosidases that hydrolyze the specific positions and types of sugar moieties in ginsenosides. Crit. Rev. Biotechnol..

[11] Jeong EB, Kim SA, Shin KC, Oh DK (2020). Biotransformation of protopanaxadiol-type ginsenosides in Korean ginseng extract into food-available compound K by an extracellular enzyme from *Aspergillus niger*. J. Microbiol. Biotechnol..

[12] Lee SJ, Lee JS, Lee E, Lim TG, Byun S (2018). The ginsenoside metabolite compound K inhibits hormone-independent breast cancer through downregulation of cyclin D1. J. Funct. Foods.

[13] Yang XD, Yang YY, Ouyang DS, Yang GP (2015). A review of biotransformation and pharmacology of ginsenoside compound K. Fitoterapia.

[14] Han Y, Sun B, Hu X, Zhang H, Jiang B, Spranger MI (2007). Transformation of bioactive compounds by *Fusarium sacchari* fungus isolated from the soil-cultivated ginseng. J. Agric. Food Chem..

[15] Zhou W, Yan Q, Li JY, Zhang XC, Zhou P (2008). Biotransformation of *Panax notoginseng* saponins into ginsenoside compound K production by *Paecilomyces bainier* sp.229. J. Appl. Microbiol..

[16] Bae SH, Lee HS, Kim MR, Kim SY, Kim JM, Suh HJ (2011). Changes of ginsenoside content by mushroom mycelial fermentation in red ginseng extract. J. Ginseng. Res..

[17] Hsu BY, Lu TJ, Chen CH, Wang SJ, Hwang LS (2013). Biotransformation of ginsenoside Rd in the ginseng extraction residue by fermentation with *lingzhi* (*Ganoderma lucidum*). Food Chem..

[18] Li Z, Ahn HJ, Kim NY, Lee YN, Ji GE (2016). Korean ginseng berry fermented by mycotoxin non-producing *Aspergillus niger* and *Aspergillus oryzae*: Ginsenoside analyses and anti-proliferative activities. Biol. Pharm. Bull..

[19] Li Z, Ji GE (2017). Ginseng fermented by mycotoxin non-producing *Aspergillus niger*: ginsenoside analysis and anti-proliferative effects. Food Sci. Biotechnol..

[20] Kim SA, Jeong EB, Oh DK (2021). Complete bioconversion of protopanaxadiol-type ginsenosides to compound K by extracellular enzymes from the isolated strain *Aspergillus tubingensis*. J. Agric. Food Chem..

[21] Shin KC, Kim TH, Choi JH, Oh DK (2018). Complete biotransformation of protopanaxadiol-type ginsenosides to 20-O-β-glucopyranosyl-20(*S*)-protopanaxadiol using a novel and thermostable β-glucosidase. J. Agric. Food Chem..

[22] Veen Pvd, Flipphi MJA, Voragen AGJ, Visser J (1991). Induction, purification and characterisation of arabinases produced by *Aspergillus niger*. Arch. Microbiol..

[23] Moussa TAA, Tharwat NA (2007). Optimization of cellulase and β-glucosidase induction by sugarbeet pathogen *Sclerotium rolfsii*. Afr. J. Biotechnol..

[24] Hanif A, Yasmeen A, Rajoka MI (2004). Induction, production, repression, and de-repression of exoglucanase synthesis in *Aspergillus niger*. Bioresour. Technol..

[25] Kim TH, Yang EJ, Shin KC, Hwang KH, Park JS, Oh DK (2018). Enhanced production of β-D-glycosidase and α-Larabinofuranosidase in recombinant *Escherichia coli* in fed-batch culture for the biotransformation of ginseng leaf extract to ginsenoside compound K. Biotechnol. Bioprocess Eng..

[26] Qu L, Ren LJ, Sun GN, Ji XJ, Nie ZK, Huang H (2013). Batch, fed-batch and repeated fed-batch fermentation processes of the marine thraustochytrid *Schizochytrium* sp.for producing docosahexaenoic acid. Bioprocess Biosyst. Eng..

